# Understanding plant–microbe interaction of rice and soybean with two contrasting diazotrophic bacteria through comparative transcriptome analysis

**DOI:** 10.3389/fpls.2022.939395

**Published:** 2022-11-18

**Authors:** Manish Ranjan Saini, Latha P. Chandran, Kalyani Makarand Barbadikar, Amitha Mithra V. Sevanthi, Gautam Chawla, Megha Kaushik, Ekta Mulani, Amol Sarjerao Phule, Rajani Govindannagari, Bandeppa Sonth, Subodh Kumar Sinha, Raman Meenakshi Sundaram, Pranab Kumar Mandal

**Affiliations:** ^1^ Indian Council of Agricultural Research (ICAR) National Institute for Plant Biotechnology, New Delhi, India; ^2^ Kalinga Institute of Industrial Technology (KIIT) School of Biotechnology, KIIT University, Bhubaneswar, India; ^3^ ICAR-Indian Institute of Rice Research, Hyderabad, India; ^4^ Division of Nematology, ICAR- Indian Agriculture Research Institute, New Delhi, India

**Keywords:** *Gluconacetobacter diazotrophicus*, Bradyrhizobium japonicum, rice, soybean, RNA-seq, nitrogen fixation

## Abstract

Understanding the beneficial plant–microbe interactions is becoming extremely critical for deploying microbes imparting plant fitness and achieving sustainability in agriculture. Diazotrophic bacteria have the unique ability to survive without external sources of nitrogen and simultaneously promote host plant growth, but the mechanisms of endophytic interaction in cereals and legumes have not been studied extensively. We have studied the early interaction of two diazotrophic bacteria, *Gluconacetobacter diazotrophicus* (GAB) and *Bradyrhizobium japonicum* (BRH), in 15-day-old seedlings of rice and soybean up to 120 h after inoculation (hai) under low-nitrogen medium. Root colonization of GAB in rice was higher than that of BRH, and BRH colonization was higher in soybean roots as observed from the scanning electron microscopy at 120 hai. Peroxidase enzyme was significantly higher at 24 hai but thereafter was reduced sharply in soybean and gradually in rice. The roots of rice and soybean inoculated with GAB and BRH harvested from five time points were pooled, and transcriptome analysis was executed along with control. Two pathways, “Plant pathogen interaction” and “MAPK signaling,” were specific to Rice-*Gluconacetobacter* (RG), whereas the pathways related to nitrogen metabolism and plant hormone signaling were specific to Rice-*Bradyrhizobium* (RB) in rice. Comparative transcriptome analysis of the root tissues revealed that several plant–diazotroph-specific differentially expressed genes (DEGs) and metabolic pathways of plant–diazotroph-specific transcripts, *viz.*, chitinase, brassinosteroid, auxin, Myeloblastosis (MYB), nodulin, and nitrate transporter (NRT), were common in all plant–diazotroph combinations; three transcripts, *viz.*, nitrate transport accessory protein (NAR), thaumatin, and thionin, were exclusive in rice and another three transcripts, *viz.*, NAC (NAM: no apical meristem, ATAF: Arabidopsis thaliana activating factor, and CUC: cup-shaped cotyledon), ABA (abscisic acid), and ammonium transporter, were exclusive in soybean. Differential expression of these transcripts and reduction in pathogenesis-related (PR) protein expression show the early interaction. Based on the interaction, it can be inferred that the compatibility of rice and soybean is more with GAB and BRH, respectively. We propose that rice is unable to identify the diazotroph as a beneficial microorganism or a pathogen from an early response. So, it expressed the hypersensitivity-related transcripts along with PR proteins. The molecular mechanism of diazotrophic associations of GAB and BRH with rice *vis-à-vis* soybean will shed light on the basic understanding of host responses to beneficial microorganisms.

## Introduction

The production of rice is proportional to the use of nitrogen in the form of chemical fertilizer, which is unfavorable from ecological and economic perspectives (Kummu et al., 2012; Yi et al., 2019). Biological nitrogen fixation (BNF) has the potential to significantly reduce the usage of artificial fertilizers in rice while reducing environmental issues. Plants associated with biological nitrogen-fixing bacteria can substantially reduce the use of chemical fertilizers in agriculture that reduces the cost of production and helps in overcoming the related environmental issues (Hardoim et al., 2008; Berg, 2009; Anitha and Thangaraju, 2010; Meenakshisundaram and Santhaguru, 2010). Diazotrophs are bacteria that can supplement biological nitrogen directly (endophytes), promote nutrient uptake and phytohormone synthesis, and impart tolerance under biotic and abiotic stress conditions, rightly called plant growth-promoting bacteria (PGPB). Endophytic colonization is the process through which endophytes enter, develop, and multiply within the host plant (Kandel et al., 2017). Apart from their association with legumes, the association of diazotrophs has also been observed with non-leguminous crop plants, e.g., *Gluconacetobacter diazotrophicus* (GAB) with sugarcane, Cameroon grass, finger millet, wetland rice, etc. (Loganathan and Nair, 2003; Muthukumarasamy et al., 2005; Rouws et al., 2010; Eskin et al., 2014; Dent, 2018); *Azospirillum* sp. with wheat (Baldani et al., 1983); *Herbaspirillum seropedicae* with maize (Baldani et al., 1986); and *Gluconacetobacter johannae* and *Gluconacetobacter azotocaptans* with coffee plants (Fuentes-Ramirez et al., 2001; Compant et al., 2010). *Bradyrhizobium japonicum* (BRH) is known to be symbiotically associated with different legume plants where it forms nodules in the roots and fixes nitrogen (Yan et al., 2016; Yuan et al., 2017). BRH also gets associated with many non-legumes including rice as endophytes and is known for its plant growth-promoting (PGPB) activity (Chaintreuil et al., 2000; Wang et al., 2018; Schneijderberg et al., 2018; Greetatorn et al., 2019). The non-nodulating endophytic GAB is associated with the rhizosphere of the plants, especially with the plants of the *Poaceae* family including sugarcane and rice (Loganathan and Nair, 2003; Muthukumarasamy et al., 2005; Rouws et al., 2010; Eskin et al., 2014; Dent, 2018). GAB has been known for its PGPB activity in several crops (Gillis et al., 1989; Muthukumarasamy et al., 2002; Govindarajan et al., 2008; Luna et al., 2012; de Souza et al., 2016). In rice, under field conditions, GAB enhances several plant growth-related parameters such as nitrogen content in the plant and in the soil, biomass, and tiller number by influencing nutrient acquisition and producing phytohormones or stress-relieving enzymes (Muthukumarasamy et al., 2005; Govindarajan et al., 2008; Dudeja et al., 2012; de Souza et al., 2016). Still, now, very few comprehensive studies on the interaction of endophytes are available, especially focusing on the beneficial interaction of cereals.

We have studied the interaction of two different diazotrophic bacteria, GAB and BRH, with rice and soybean under low-nitrogen conditions for a better understanding of the differential host response. GAB and BRH were contrasting, as GAB does not induce nodule formation while BRH induces nodule formation in the legumes. Starting from changes in bacterial colonization pattern, root and shoot morphology, and hypersensitivity reactions, we made a comparative transcriptome profiling of the root tissues after inoculation. The expression profile of the critical early responsive genes was studied in detail at five time points after inoculation. This information would offer a possible approach for identifying the missing link between symbiotic nitrogen-fixing legumes and non-legume plants and for developing acceptable techniques for using BNF systems to provide an efficient nitrogen supply to rice crops.

## Materials and methods

### Plant material, bacterial cultures, and experimental setup

Rice seeds (variety TN1) and bacterial cultures GAB PAL5 (MTCC 1224) and BRH (MCC 2152) were obtained from ICAR-Indian Institute of Rice Research, Hyderabad, Telangana, India, and soybean seeds (variety SL-1028) were obtained from the Division of Genetics, ICAR-Indian Agriculture Research Institute, Pusa, New Delhi, India. The TN1 variety was considered for this study because it is one of the most disease- and pest-susceptible varieties of rice inoculation. The soybean variety, SL-1028, was considered, as it is one of the recently released varieties of soybean.

Uniform healthy seeds were surface sterilized using 0.5% sodium hypochlorite for 1 min followed by five thorough washes of sterile double-distilled water. The sterilized seeds were kept for germination in the dark at 28°C on germination paper soaked with sterile distilled water. Germinated seedlings of 2–4 cm (3 days in the case of rice and 4 days in the case of soybean) were transferred into pots (4-inch diameter and 150-ml media), considering five individual plants per pot, and kept in the culture room with controlled light (16 h, 1,000 lux), temperature (28°C ± 2^°^C), and relative humidity (80%). Seedlings were grown in hydroponics using modified Yoshida media (Yoshida et al., 1976) for rice and Hoagland salt mixer (Hoagland and Arnon, 1950) for soybean for a period of 15 days. We used zero nitrogen-containing media, and according to the requirement of nitrogen, we mixed ammonium nitrate salt into them. The medium contained 8.0 mM of nitrogen from ammonium nitrate salt for both rice and soybean for optimum growth. The hydroponics solution was replaced with fresh medium every third day. After the 15th day, the medium was replaced with fresh medium containing 0.08 mM of nitrogen. GAB and BRH cultures were added to the media to obtain a concentration of 10^8^ CFU/ml (culture medium concentration), while seedlings growing in media without the culture served as control. Root tissues were collected from rice and soybean plants sequentially at five time points, *viz.*, 24, 48, 72, 96, and 120 h after inoculation (hai). The root samples of the five time points were pooled together in two biological replicates. The samples were stored at -80°C freezers after snap freezing in liquid nitrogen for total RNA isolation and further downstream processing.

The root tissue samples inoculated with GAB, BRH, and control will be hereafter mentioned as RG (Rice-GAB), RB (Rice-BRH), RC (Rice-Control), SG (Soybean-GAB), SB (Soybean-BRH), and SC (Soybean-control).

### Seedling morphology

The length (cm) (root and shoot) and weight (mg) (fresh and dried) of the rice and soybean seedlings were measured in three biological replicates using a metric scale and weighing balance, respectively. The shoots and roots were dried at 50°C, and the dry weights were estimated using the constant weight method. The root system architecture (RSA) of the seedling was recorded in WinRhizo software (Regent Instruments Canada Inc. 2013) at 400 dots per inch (dpi) using a flatbed root scanner (Epson v700, Seiko Epson Corporation, Japan) according to the procedure described by Sinha et al. (2018).

### Colonization by scanning electron microscopy

Scanning electron microscopy (SEM) was carried out to assess the colonization ability of the bacterial cultures at 120 hai. We have cut the entire root sample into 1-cm pieces and 7–8 pieces were processed, of which four randomly selected pieces were observed under the microscope. Before processing for SEM, the roots were fixed overnight at room temperature in glutaraldehyde (3% in 0.1 M phosphate buffer, pH 7.2) followed by 6 h in 1% osmium tetraoxide (aqueous). The samples were then dehydrated with a series of 40%, 50%, 60%, 70%…100% ethanol for 30 min each. The samples were then dried using CO_2_ gas and were coated with a 40-μM layer of gold by using an automated sputter coater and examined for bacterial colonization using SEM (VEGA3 TESCAN, Czechia). The entire 1-cm portion of each sample was observed, and we found the random distribution of bacteria on the root surface. We have done different levels of magnification for different samples. When we did not see any bacterial colonization, we observed in lower magnification to cover a larger area; when we saw some colonization, we enlarged that particular area for confirmation.

### Antioxidant enzyme peroxidase assay

Peroxidase (POD; EC 1.11.1.7) activity of frozen root samples was measured as per the modified method of using guaiacol as substrate (electron donor) (Castillo et al., 1984; Prakash et al., 2016). The root samples (100 mg) were homogenized using tissue lyzer (EzLyzer, Genetix, Biotech Asia, Pvt. Ltd.) in 300 µl extraction buffer (0.1 M phosphate buffer and 0.1 mM EDTA, pH 7.0). Lysates were centrifuged for 20 min at 15,000 × g at 4°C temperature, and the supernatants were collected and stored for assay. The 3-ml reaction mixture contained 16 mM guaiacol, 2 mM H_2_O_2_, 50 mM phosphate buffer (pH 6.1), and 0.1 ml enzyme extract. The change in optical density (OD) of the reaction mixture was recorded at 436 nm for a period of 3 min in time scan mode using a UV-Visible spectrophotometer (Thermo Scientific, USA). The result was presented as a change in OD per min/g fresh weight.

### RNA extraction and illumina sequencing

Total RNA from root tissue was extracted using PureLink™ RNA mini Kit (Invitrogen Bio Services India Pvt. Ltd.). RNA quality and quantity were checked by agarose gel electrophoresis and NanoDrop spectrophotometer (Thermo Scientific, USA). For RNA sequencing (RNA-seq), RNA from the root samples of the five time points (24, 48, 72, 96, and 120 hai) was extracted and pooled in equimolar concentration. For each plant–microbe combination, total RNA was isolated from two biological replicates independently. As a result, 12 different libraries labeled as RG, RB, RC, SG, SB, and SC were constructed and sequenced according to the manufacturer’s protocol, as mentioned in Kaushik et al. (2020), using paired-end Illumina HiSeq 2500. All of the raw sequence read archives (SRAs) were submitted to National Center for Biotechnology Information (NCBI) with accession number PRJNA472691.

### RNA sequencing data processing, read alignment, and analysis

The quality of paired-end raw reads was checked by using the FastQC tool (http://www.bioinformatics.bbsrc.ac.uk/projects/fastqc/). In the case of rice, 88.48% and, in the case of soybean, 89.36% of high-quality clean reads (with an average Phred quality score ≥20) were obtained, which were further used for downstream analysis. High-quality reads obtained were mapped onto the reference genome (*Oryza sativa japonica* IRGSP-1.0.34 and Glycine_max.V1.0) by using TopHat (v2.1.0) software. The mismatch parameter was set to 2.0, and other parameters such as read gap length, read edit distance, maximum deletion length, maximum multi-hits, and minimum and maximum intron length were set to default. Only filtered reads were further processed for analyzing the mapping status of RNA-seq data. The following comparative analysis was made for the plant–microbe interactions to identify the differentially expressed transcripts (**Table 1**).

**Table 1:** Different comparative analyses for plant–microbe interactions.

Treatment 1	Treatment 2	Comparison b/w
RG	RC	RG × RC
RB	RC	RB × RC
RB	RG	RB × RG
SG	SC	SG × SC
SB	SC	SB × SC
SB	SG	SB × SG
SC	RC	SC × RC
SG	RG	SC × RG
SB	RB	SC × RB

RG, Rice-Gluconacetobacter; RB, Rice-Bradyrhizobium; RC, Rice-Control; SG, Soybean-Gluconacetobacter; SB, Soybean-Bradyrhizobium; SC, Soybean-control.

### Differential gene expression

The expression analysis for differentially expressed genes (DEGs) was carried out using the DESeq2 v1.24.0 R package (each treated sample normalized with control). Size factor calculations in the DESeq tool were utilized for reducing the sequencing (uneven library size/depth) bias among the samples and library normalization. The number of DEGs for distinct pairings of biologically significant genotype comparisons was determined using a rigorous comparison at a p-value of 0.05, log2 fold change 1 (for upregulation) and -1 (for downregulation).

### Cross-species differential gene expression analysis

For gene expression analysis between two different species, evolutionarily conserved transcripts need to be considered. The orthologous matrix (OMA) browser was used to filter 1:1 orthologous genes between two species according to the different combinations shown in **Table 1**.

### Functional analysis

The BLAST program from the Uniprot database was used for the annotation of transcripts against *O. sativa.* Blast2GO suite (software) and AgriGO (http://bioinfo.cau.edu.cn/agriGO/analysis.php) programs were utilized for different functional analyses such as Kyoto Encyclopedia of Genes and Genomes (KEGG) pathways and Gene Ontology (GO), respectively, with the parameter setting mentioned earlier in Kaushik et al. (2020).

### Quantitative real-time PCR

Quantitative real-time PCR (qRT-PCR) was carried out for validation of RNA-seq data and expression analysis of key genes at five time points, i.e., 24 hai, 48 hai, 72 hai, 96 hai, and 120 hai. For validation of RNA-seq data, we have used the pooled RNA samples from the five time points as in the case of RNA-seq by Illumina. For key gene expression analysis and validation at the five time points, we used RNA samples of respective time points, i.e., 24 hai, 48 hai, 72 hai, 96 hai, and 120 hai. Control samples from each time point were included in the validation. Primer designing, total RNA extraction, and cDNA preparation were carried out in the same way as mentioned in Kaushik et al. (2020). Rice actin (*LOC_Os03g50885*) and soybean actin (*LOC100781831*) were used as internal control. Primer sequences used in this study for qRT-PCR have been provided in **Supplementary Material S1**.

### Statistical analysis

All of the experiments (morphological parameters, RSA, and enzyme assay) were conducted in a complete randomized design (CRD) with three biological replications (three technical replicates per biological replicate). Error bars were mentioned by calculating the Standard Errors of Means wherever applicable.

## Results

### *Gluconacetobacter diazotrophicus* and *Bradyrhizobium japonicum* root colonization and seedling morphology

**Figure 1:**
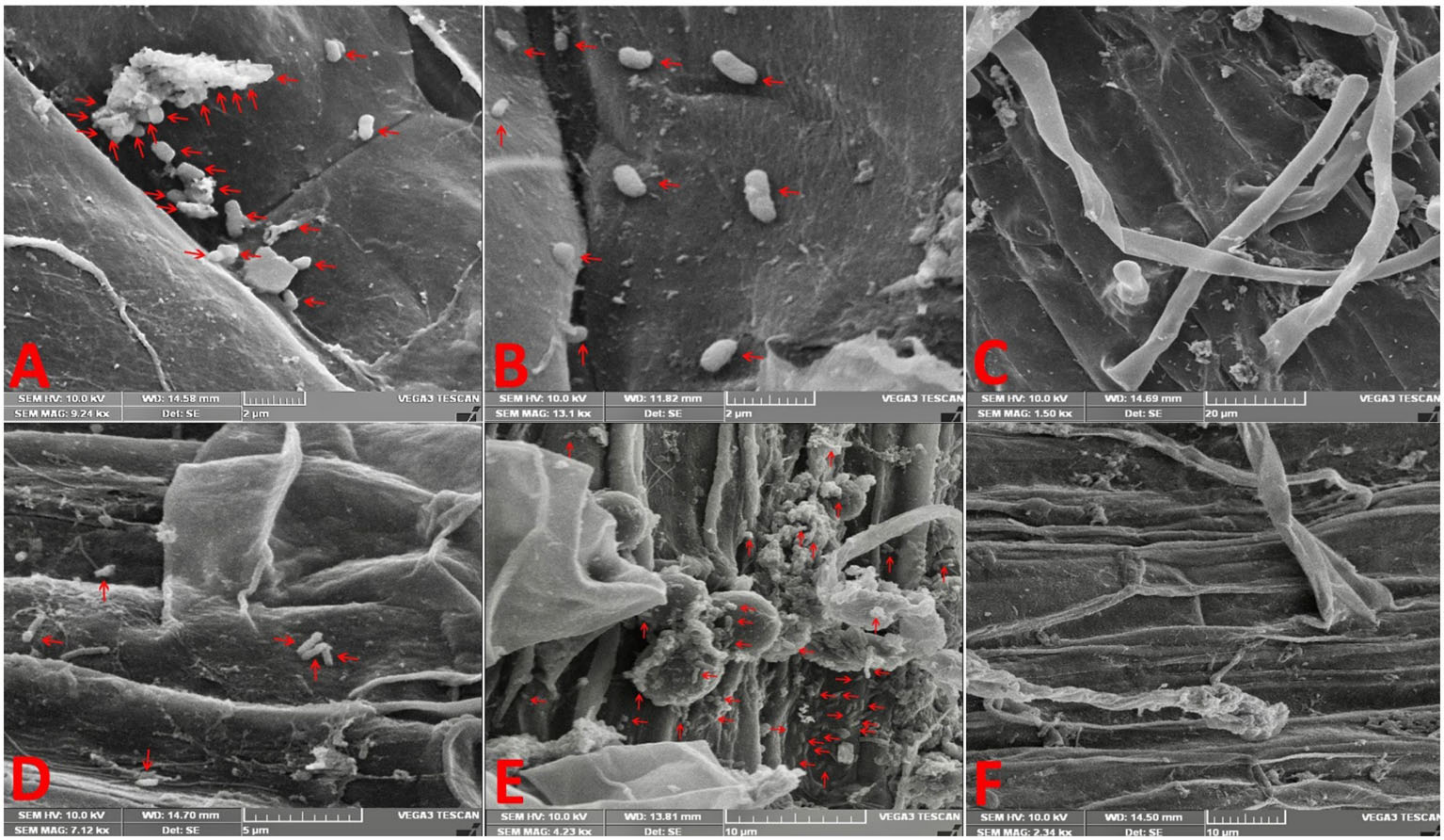
Electron micrographs reveal the adherence of bacteria on the root surface at 120 h after inoculation. **(A)** Rice root inoculated with *Gluconacetobacter diazotrophicus* (GAB), **(B)** rice root inoculated with Bradyrhizobium japonicum (BRH), **(C)** rice control (uninoculated root), **(D)** soybean root inoculated with GAB, **(E)** soybean root inoculated with BRH, and **(F)** soybean control (uninoculated root).

SEM of inoculated rice and soybean roots revealed that both bacteria adhered to the surface of the roots mainly at 120 hai (**Figures 1A–F**). We observed that a much greater number of bacteria adhered to the root surface of rice when inoculated with GAB and of soybean when inoculated with BRH in comparison to the other combinations (RB and SG, respectively). Bacterial growth was not observed in the control treatment. We did not notice any significant colonization up to 96 hai (**Supplementary Figures S1A–F**).

**Figure 2:**
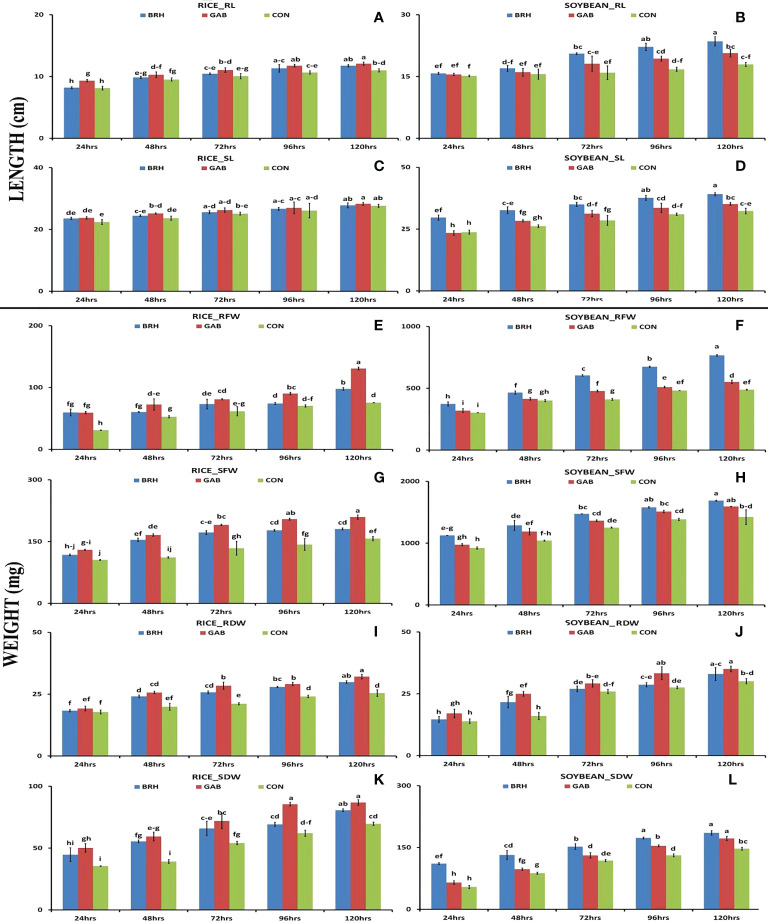
Graphs showing the morphological parameters of rice and soybean roots at five time points after inoculation with *Gluconacetobacter diazotrophicus* (GAB) and Bradyrhizobium japonicum (BRH). **(A)** Rice_RL (root length); **(B)** Soybean–RL; **(C)** Rice_SL (shoot length); **(D)** Soybean_SL; **(E)** Rice_RFW (root fresh weight); **(F)** Soybean_RFW; **(G)** Rice_RDW (root dry weight); **(H)** Soybean_RDW; **(I)** Rice_SFW (shoot freshweight); **(J)** Soybean_SFW; **(K)** Rice_SDW (shoot dry weight); **(L)** Soybean_SDW. Small letter on the top of the bar indicates the critical differences. Same letters indicate statistically non-significant and different letters indicate statistically significant.

We observed differentiation in seedling growth parameters of rice and soybean cultivars at the five time points following inoculation with diazotrophs (**Figures 2A–L**). In both plants, there was a gradual and significant increase in all of the recorded morphological parameters [root length (RL), shoot length (SL), root fresh weight (RFW), shoot fresh weight (SFW), root dry weight (RDW), and shoot dry weight (SDW)] from 24 hai to 120 hai period in all of the conditions. In the case of RL (**Figure 2A**) and SL (**Figure 2C**) of rice, the increases were larger with GAB in comparison to BRH. However, in the case of soybean (**Figures 2B, D**), the increase with BRH inoculation was significant at all of the time points for SL and 72 hai onward for RL. SFW, SDW, RFW, and RDW (**Figures 2E–L**) were significantly increased at 120 hai with both diazotrophs in both rice and soybean when compared to the control. A uniform trend of change was observed in these morphological parameters after inoculation up to 120 hai, which was plant-specific. In the case of rice, all of the parameters were significantly increased by GAB inoculation; in the case of soybean, a significant increase was induced by BRH at most time points with a few exceptions. However, interestingly, as an exception, RDW was much higher in soybean inoculated with GAB; BRH inoculation, on the other hand, resulted in a higher increase in all morphological parameters.

### Enhanced root-related traits upon diazotrophic associations of rice and soybean

**Figure 3:**
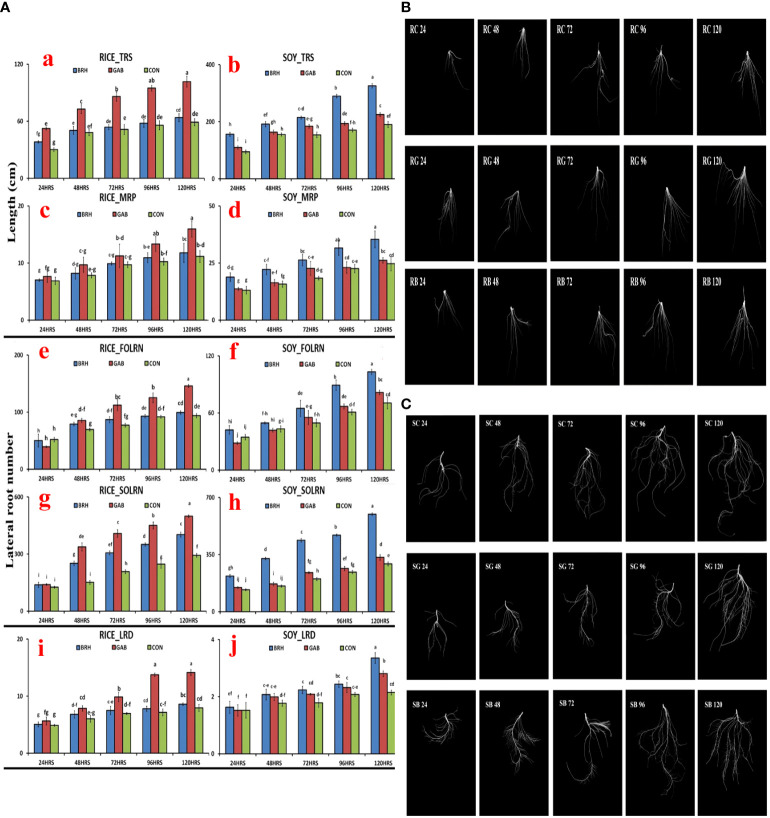
**(A)** Root system architecture (RSA) parameters of the two crops, rice and soybean, after inoculation with Gluconacetobacter diazotrophicus (GAB) and Bradyrhizobium japonicum (BRH); (a) Total root size (TRS) of rice; (b) TRS of soybean; (c) Main root path length of seminal root (MRP) of rice; (d) MRP of soybean; (e) first-order lateral root number (FOLRN) of rice; (f) FOLRN of soybean; (g) second-order lateral root number (SOLRN) of rice; (h) SOLRN of soybean; (i) lateral root density (LRD) of rice; (j) LRD of soybean. **(B)** Changes in the RSA of rice with different diazotrophs at different time points (24–120 h after inoculation). First row–Rice-control; Second row–Rice-GAB; Third row–Rice-BRH. **(C)** Changes in the RSA of soybean with different diazotrophs at different time points (24–120 h after inoculation). First row–Soybean-control; Second row–Soybean-GAB; Third row–Soybean-BRH. Small letter on the top of the bar indicates the critical differences. Same letters indicate statistically non-significant and different letters indicate statistically significant.

Parameters of the RSA, *viz.*, total root size (TRS), main root path length of the seminal root (MRP), first-order lateral root number (FOLRN), second-order lateral root number (SOLRN), and lateral root density (LRD), of rice and soybean were measured after inoculation with GAB and BRH (**Figures 3A–J**). The scanned images of the RSA parameter at different time points of both rice and soybean after inoculations are shown in **Figures 3B, C**, respectively. Two very clear observations from the result of RSA were noticed, as follows: 1) all of the major parameters of RSA recorded in the study increased when inoculated with either of the diazotrophs from 48 hai onward; 2) in the case of rice, the increase in RSA parameters was significantly higher when inoculated with GAB, and in soybean, when inoculated with BRH. The complete data of all five RSA parameters at the five time points are provided in **Supplementary Material S2**.

### Root-related differential peroxidase activity during the interaction

**Figure 4:**
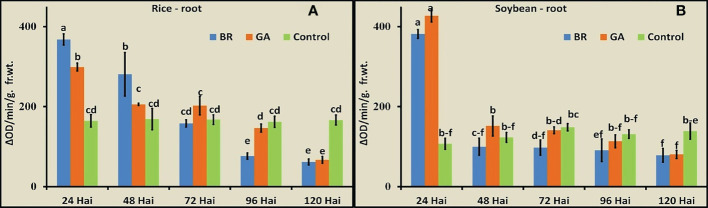
Peroxidase activity at the five time points after inoculation with *Gluconacetobacter diazotrophicus* (GAB) and *Bradyrhizobium japonicum* (BRH). **(A)** Rice root, **(B)** soybean root. Small letter on the top of the bar indicates the critical differences. Same letters indicate statistically non-significant and different letters indicate statistically significant.

The activity of POD was observed in roots of rice and soybean after inoculation with diazotrophs at the five time points (**Figures 4A, B**). In both cases, immediately after inoculation with the diazotrophs, the POD activity increased to a significantly higher level. The soybean-BRH and rice-GAB combinations showed the maximum values of POD, and the activity gradually decreased in rice and soybean. The decrease was very sharp, soon after the rise (from 24 hai to 48 hai), in the case of soybean, whereas the decrease was gradual over a time period in the case of rice.

### Transcriptomic profiles of rice and soybean roots upon inoculation with *Gluconacetobacter diazotrophicus* and *Bradyrhizobium japonicum*

The data emanated by the sequencing of 12 RNA-seq libraries were deployed for transcriptomic analysis and differential gene expression. Approximately 416 and 447 million raw reads were obtained from rice and soybean, respectively, with at least 68 million reads for each sample. After a quality check of sequencing data, a major proportion (88.48% in the case of rice and 89.36% in the case of soybean) of high-quality clean reads (with an average Phred quality score ≥20) were mapped to rice and soybean reference genomes. The assembly of mapped reads resulted in the identification of 66,220 and 103,715 transcripts in rice and soybean, respectively (**Supplementary Material S3**). A total of 63, 113, 779, 2,509, 2,158, 3,925, 1,568, 1,604, and 1,619 DEGs were found in RG/RC, RB/RC, RB/RG, SG/SC, SB/SC, SB/SG, SC/RC, SG/RG, and SB/RB, respectively (**Supplementary Materials S4, S5**). Furthermore, important DEGs in all possible combinations are provided in **Supplementary Material S6**.

We have also identified the orthologous genes between rice and soybean. This helped us to identify the diazotroph-specific gene expression in both plant species (**Supplementary Material S7**). When we compared orthologous DEGs from control and GAB (**Supplementary Figure S2**), we found that 125 DEGs were exclusive in control and 161 were exclusive in GAB inoculation and 1,443 DEGs were expressed regardless of the inoculation/inoculation by GAB. Similarly, when we analyzed DEGs from control and BRH (**Supplementary Figure S2**), we found that 122 DEGs were exclusive in control, 173 DEGs were exclusive in BRH inoculation, and 1,446 DEGs were common. The majority of the genes were differentially expressed in both crops (based on orthologous gene study) regardless of any inoculation/inoculation by either of the bacteria. However, from the above data (**Supplementary Figure S2**), we found 73 DEGs regardless of diazotrophic inoculation, whereas 88 were GAB-specific and 100 were BRH-specific.

### Expression analysis of important differentially expressed genes after diazotroph inoculation

**Figure 5:**
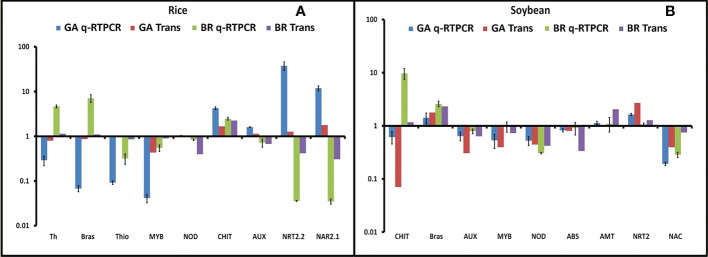
Validation of all 12 differentially expressed genes (DEGs). **(A)** Fold change of all nine selected DEGs in rice was plotted against the qRT-PCR result. **(B)** Fold change of all nine selected DEGs in soybean was plotted against the qRT-PCR result. Abbreviations: Th, thaumatin; Brs, brassinosteroid receptor; Thi, thionin; NOD, Early Nodulin36A protein; CHI, chitinase; AUX, auxin response factor; NRT, nitrate transporter; NAR, nitrate transport accessory protein.

To validate the RNA-seq results, qRT-PCR was performed in both rice and soybean from all of the combinations of plant–microbe interaction. In our study, we found the 12 most promising genes based on their differential expression and functional annotation. Six genes were common, and three each were specific to rice and soybean. The selected DEGs were related to defense, signaling, stress response, and nitrogen acquisition, *viz*., thaumatin, brassinosteroid, thionin, nodulin, chitinase, auxin, nitrate transporter, and nitrate transport accessory protein. Fold change observed through qRT-PCR was consistent with the changes in expression level measured by RNA-seq (**Figures 5A, B**).

**Figure 6:**
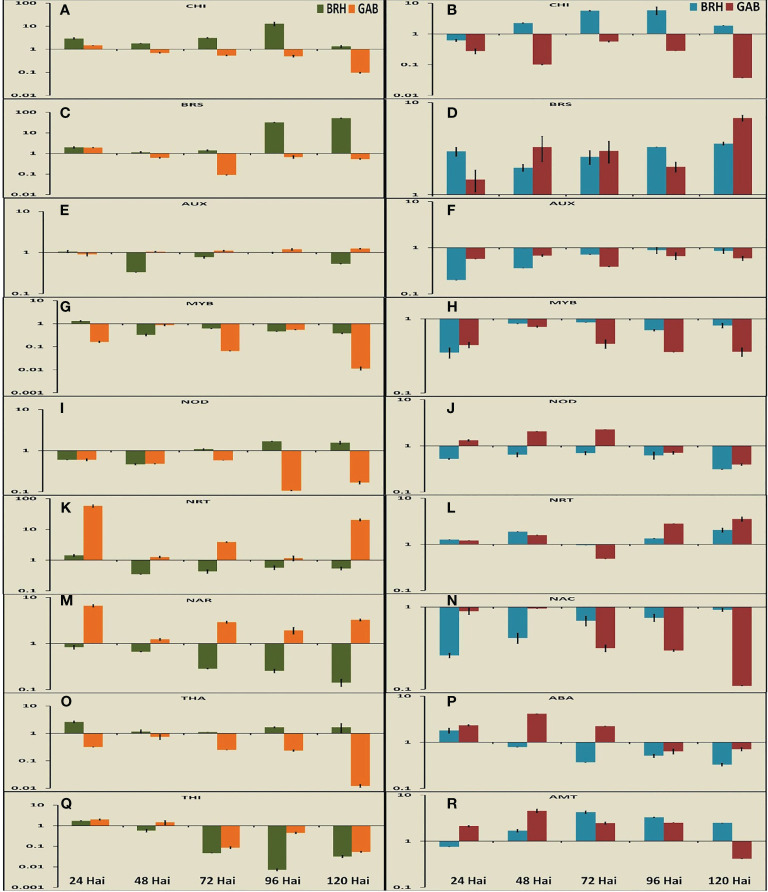
Expression pattern of important differentially expressed genes (nine in rice and nine genes in soybean) at different time points after inoculation with the diazotrophs. **(A)** Expression pattern of chitinase at different time points in rice. **(B)** Expression pattern of chitinase at different time points in soybean. **(C)** Expression pattern of brassinosteroid insensitive 1-associated receptor kinase at different time points in rice. **(D)** Expression pattern of brassinosteroid receptor at different time points in soybean. **(E)** Expression pattern of auxin response factor at different time points in rice. **(F)** Expression pattern of auxin-inducible protein at different time points in soybean. **(G)** Expression pattern of MYB transcription factor at different time points in rice. **(H)** Expression pattern of MYB transcription factor at different time points in soybean. **(I)** Expression pattern of *Medicago truncatula* nodulins at different time points in rice. **(J)** Expression pattern of Early Nodulins36A protein at different time points in soybean. **(K)** Expression pattern of high-affinity nitrate transporter (NRT) at different time points in rice. **(L)** Expression pattern of high-affinity NRT at different time points in soybean. **(M)** Expression pattern of nitrate transport accessory protein (NAR) at different time points in rice. **(N)** Expression pattern of NAC transcription factor at different time points in soybean. **(O)** Expression pattern of thaumatin at different time points in rice. **(P)** Expression pattern of abscisic acid at different time points in soybean. **(Q)** Expression pattern of thionin at different time points in rice. **(R)** Expression pattern of ammonium transporter at different time points in soybean.

Nine DEGs each in rice and soybean were analyzed in root tissues using qRT-PCR at the five time points after inoculation (**Figures 6A–R**). In rice, four DEGs were related to defense, three were related to nodulation and nitrogen transport, and two were related to signaling. We have studied three DEGs related to defense, three related to nodulation and nitrogen transport, and three related to signaling in the case of soybean.

The expression of thaumatin (*Os03G0661600*), a gene encoding pathogen response protein [Pathogenesis-Related (PR) Protein 5] was specific to rice and reduced gradually from 24 hai to 120 hai in both RG and RB (**Figure 6O**). However, the expression was significantly higher in RB than that in RG. The expression of the class III chitinase gene in rice (Miyamoto et al., 2012) (*OsChib3a*: *Os01G0660200*) and chitinase 1 gene in soybean (Renner and Specht, 2012) (*GmChitI-1: GLYMA02G04820*), representing proteins involved in pathogen response, decreased at 120 hai after attaining the peaks at different time points for different plant–bacteria combinations in both rice and soybean (**Figures 6A, B**). As such, BRH induced the expression of chitinase at a much higher level than that induced by GAB in both plant species. The expression of chitinase was also increased up to 96 hai in the case of BRH, whereas the peak expression in the case of GAB was at 24 hai (rice) and 72 hai (soybean). The last pathogen response-related protein examined, thionin (Ji et al., 2015) (*Osthi1: Os06G0509900*), was differentially expressed only in rice, and its expression went down sharply from 24 hai with both GAB and BRH inoculation (**Figure 6Q**). Induction of stress-related genes encoding abscisic acid (ABA) stress ripening-like protein (*GLYMA20G30720*) was significant only in the case of soybean (**Figure 6P**), and the change of expression was high during the initial stages after inoculation (24 hai in case of BRH and 48 hai in case of GAB).

Two genes, which encode important transcription factors, NAC and MYB, were differentially expressed. *GmNAC1* (*GLYMA04G38560*; Pinheiro et al., 2009) was differentially expressed only in soybean (**Figure 6N**), whereas the MYB-encoding gene was found to be differentially expressed in both species [*OsMYBS1*: *Os11G0700500* in rice (Chen et al., 2017) and *GmMYB76*: *GLYMA02G01300* (Liao et al., 2008) in soybean] (**Figures 6G, H**). The expression of *GmNAC1*: *GLYMA04G38560* was reduced from the peak at 48 hai to a minimal level at 120 hai in the case of GAB. However, BRH-induced expression of *GmNAC1*: *GLYMA04G38560* continued to increase up to 120 hai. In the case of rice, *OsMYBS1*: *Os11G0700500* expression was at its peak at 24 hai with BRH and 48 hai with GAB, whereas in the case of soybean, *GmMYB76*: *GLYMA02G01300* expression was at its highest level at 72 hai with BRH and 48 hai with GAB.

Genes involved in the response to an important phytohormone, namely, auxin [*OsARF10: Os04G0519700* auxin response factor 10 in rice (Xu et al., 2017) and *GmAUX2 11: GLYMA02G16090* auxin-inducible protein in soybean], and in the response to another phytohormone, brassinosteroid [*OsI-BAK1: Os11G0514500* brassinosteroid insensitive 1- associated receptor kinase in rice (Ye et al., 2021) and *GmBri1b: GLYMA04G39610* brassinosteroid receptor in soybean (Peng et al., 2016)], were differentially expressed with both bacterial inoculations (**Figures 6C–F**). Auxin and brassinosteroids are responsible for signaling in different processes. Although we found an almost similar trend of expression of important DEGs during 24 hai to 120 hai, in the case of these two genes, the expression was completely contrasting in the two crops in response to the inoculation of two different diazotrophs at 120 hai. The genes for the auxin response factor (*OsARF10: Os04G0519700*) and the auxin-inducible protein (*GmAUX2 11: GLYMA02G16090*) were differentially expressed in both rice and soybean, respectively. In the case of rice, expression remained at the same level in response to inoculation with both GAB and BRH at 24 hai but increased with GAB and decreased with BRH inoculation at 120 hai. In the case of soybean, the level of expression of the auxin-inducible protein gene was considerably low with BRH at 24 hai but increased to a considerable level at 120 hai. The exact opposite trend was observed in the case of the brassinosteroid receptor. Gene expression was almost similar at 24 hai in both rice (*OsI-BAK1: Os11G0514500*) and soybean (*GmBri1b: GLYMA04G39610*). However, rice and soybean had a much higher expression with BRH and GAB, respectively, at 120 hai.

The other four significant DEGs found in the study were related to nitrogen metabolism. Genes encoding a high-affinity nitrate transporter (NRT2) and two different genes linked to nodulation, a soybean early nodulin gene (*GmENOD36a*; Kouchi and Hata, 1993; Ott et al., 2009) and the rice homolog of a nodulin from *Medicago truncatula*, *MtN21* (Denancé et al., 2014), *OsMtN21*: *Os05G0493800*, were differentially expressed (**Figures 6I–L**). Nodulin (*OsMtN21: Os05G0493800* in rice and *GmENOD36A* in soybean) gene expression was downregulated more with time when rice was inoculated with GAB and soybean with BRH. High-affinity nitrate transporter *OsNRT2.2: Os02G0112600* in rice (Yan et al., 2011) and *GLYMA13G39850* in soybean, similar to *Arabidopsis NRT2.*4 (Kiba et al., 2012), was also differentially expressed in both plant species, where GAB has upregulated the expression to a significantly higher level at 24 hai in rice and thereafter sharply reduced its expression, whereas the gene expression was downregulated throughout the entire period after BRH inoculation. The nitrate transport accessory protein *OsNAR2.1: Os02G0595900* (Yan et al., 2011) was differentially expressed only in rice, and the gene expression was gradually downregulated in RB, whereas it was upregulated in RG (**Figure 6M**). Ammonium transporter *GmAMT2*: *GLYMA18G43540* (Sohlenkamp et al., 2002) was found differentially expressed only in soybean, and with both inoculations, there was an increase, and the peak was found at 48 hai (GAB) to 72 hai (BRH) (**Figure 6R**).

### Gene ontology

The DEGs were classified into three functional groups: biological process (BP), molecular function (MF), and cellular component (CC) (**Supplementary Material S8**). It enabled us to understand whether the DEGs identified were functionally coordinated in response to microbial interactions. We have also identified a few commonly enriched GO terms between different combinations of plant–microbe interactions. The GO term “multicellular organism development” (GO:0007275), “integral component of membrane” (GO:0016021), and “oxidoreductase activity” (GO:0016449) of BP, CC, and MF, respectively, was common between RG and RB inoculation. Similarly, 54, 20, and 34 GO terms of MF, CC, and BP, respectively, were common between SG and SB inoculation (**Supplementary Material S9**).

### Pathways enriched during interaction

To understand the biological functions of DEGs further, 63 DEGs from RG/RC and 113 DEGs from RB/RC were mapped onto the KEGG database to know the significantly enriched pathway. Five KEGG pathways, *viz.*, biosynthesis of secondary metabolite (*osa01110*), phenylpropanoid biosynthesis (*osa00940*), plant–pathogen interaction (*osa04626*), flavonoid biosynthesis (*osa00941*), and Mitogen activated protein kinase (MAPK) signaling pathway (*osa04016*), were significantly enriched in RG/RC (**Supplementary Material S10**). Whereas in RB/RC, six KEGG pathways, *viz*., biosynthesis of secondary metabolites (*osa01110*), phenylpropanoid biosynthesis (*osa00940*), plant hormone signal transduction (*osa04075*), flavonoid biosynthesis (*osa00941*), nitrogen metabolism (*osa00910*), and flavone and flavone biosynthesis (*osa00944*), were enriched (**Supplementary Material S10**). In the case of soybean, the number of enriched KEGG pathways was higher (**Supplementary Material S10**), which included the pathways observed in rice, mainly related to signaling, nitrogen metabolism, plant–pathogen interaction, and biosynthesis of secondary metabolites, phenylpropanoids and flavonoids.

### Functional analysis of orthologous differentially expressed genes

**Figure 7:**
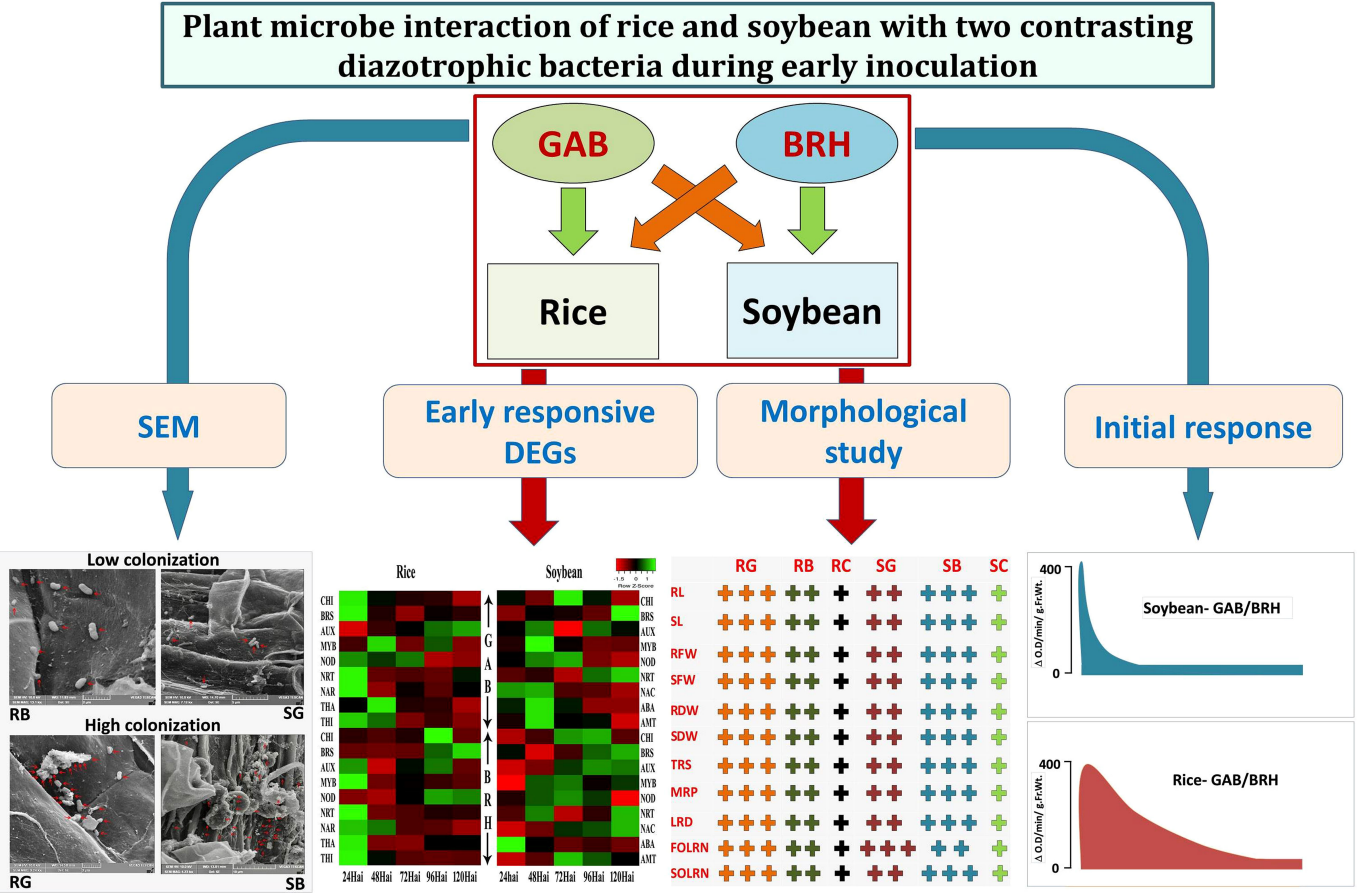
Overall effect of BRH and GAB inoculation in rice and soybean. SEM shows that BRH colonizes more in soybean root and GAB colonizes more in rice root. Hypersensitive reaction: Change in peroxidase activity at the different time points in the root tissues of rice and soybean after inoculation with BRH and GAB. Morphological study: “+” symbol denoted the effect of BRH and GAB inoculations on different parameters: “+”: normal, “+ +”: high, and “+ + +” very high. Early responsive DEGs: Pattern of highly responsive DEGs during 24–120 h after inoculations. GAB, *Gluconacetobacter diazotrophicus*; BRH, *Bradyrhizobium japonicum*; SEM, scanning electron microscopy; RB, Rice-*Bradyrhizobium*; RG, Rice-*Gluconacetobacter*; SB, Soybean-*Bradyrhizobium*; SG, Soybean *Gluconacetobacter*; NRT, high-affinity nitrate transporter; NAR, nitrate transport accessory protein; BRS, brassinosteroids; THI, thionin; NOD, nodulin; ABA, abscisic acid; AMT, ammonium transporter; RL, root length; SL, shoot length; RFW, root fresh weight; SFW, shoot fresh weight; RDW, root dry weight; SDW, shoot dry weight; TRS, total root size; MRP, main root path length of seminal root; LRD, lateral root density; FOLRN, first-order lateral root number; SOLRN, second-order lateral root number.

From the orthologous DEGs, we observed five, seven, and eight major enriched GO terms in “biological process,” “cellular components,” and “molecular function,” respectively. In rice and soybean, orthologous DEGs were 284 in control (RC and SC), 289 in GAB inoculation (RG and SG), and 302 in BRH inoculation (RB and SB) involved in biological processes. Similarly, 905, 930, and 915 DEGs were involved in cellular components, and 860, 874, and 904 were involved in molecular function in the case of control (RC and SC), GAB (RG and SG), and BRH inoculations (RB and SB), respectively (**Supplementary Material S9**). Several orthologous DEGs were significantly classified under different KEGG pathways in control (RC and SC), BRH inoculation (RB and SB), and GAB inoculation (RG and SG). Most of the pathways in all three combinations were common except for diterpenoid biosynthesis; vitamin B6 metabolism and nicotinate and nicotinamide metabolism were found in control (RC and SC) and BRH inoculation (RB and SB). Arachidonic acid metabolism pathways were exclusively present in control and were not observed in any of the diazotrophic inoculations. Cutine, suberine, and wax biosynthesis pathways were exclusively present in the case of BRH inoculations (RB and SB). However, no major pathways were exclusive for GAB inoculation (RG and SG). Details about KEGG pathways, using orthologous DEGs, have been presented in **Supplementary Material S10**. The overall representation of the results has been graphically presented (**Figure 7**).

## Discussion

With an aim to investigate the molecular mechanisms operating under beneficial root associations of the two endophytes differing in their nature of fixing nitrogen in cereal rice *vis-à-vis* legume soybean, we selected the bacterial spp., i.e., GAB and BRH. Both GAB and BRH are endophytic diazotrophic PGPB, one is a non-nodulating nitrogen fixer while the other one is a nodulating symbiotic nitrogen fixer, respectively (Loganathan and Nair, 2003; Muthukumarasamy et al., 2005; Rouws et al., 2010; Eskin et al., 2014; Dent, 2018; Madan et al., 2022). Rice is a model plant system to study the plant–microbe interaction, and studies have been documented with GAB (Rouws et al., 2010; Meneses et al., 2011; Alquéres et al., 2013). Similarly, reports have been documented for BRH interaction in soybean. Here, the selection of cereal rice and legume soybean was done purposefully to understand the early interaction dynamics of host–bacteria interaction.

The beneficial associations and the interaction studies are very much essential to have insights into the molecular responses of hosts during plant growth promotion. Additionally, the molecular mechanism and responsive genes involved in non-leguminous plant interactions, especially rice, and diazotrophic PGPB are not yet known. In this study, we have grown soybean and rice seedlings in nitrogen-deficient hydroponic conditions and inoculated with these diazotrophs. We focused specifically on the roots and executed transcriptome sequencing, as the roots are the very first organs to perceive the signals from the bacteria.

In our study, we have pooled the samples in two replications to have a representation of five data points. Two biological replicates have been used and cited widely (Xu et al., 2015; Yang et al., 2015; Ding et al., 2017; Sun et al., 2021; Li et al., 2022) for transcriptome analysis especially when it is a reference-based transcriptome like that of rice.

To capture the initial interactive transcriptome, we have selected five data points. Pooling the samples at short intervals would let us know the complete transcriptome until the last point of sampling. The transcriptome is spatiotemporal (very well time-specific), and the transcripts need to be captured at particular time points for understanding the patterns of gene expression (Kukurba and Montgomery, 2015; Takele et al., 2020). Pooling is one of the strategies for transcriptome analysis given that the data are supported by the number of reads, the number of pooling samples, the coverage, and the depth of transcriptome sequencing.

We have generated good enough data with appropriate sequencing reads and depth (transcriptomic coverage). We have used accordingly a strong and one of the most reliable tools for expression analysis, DESeq2, that is based on a model using the negative binomial distribution with stringent parameters (Love et al., 2017). DESeq2 allows for the estimation of dispersion of counts of each group, and accordingly, we have executed differential gene expression analysis using robust statistical tests. Moreover, while running the tool, we have taken care of the outliers as well and taken the data that were dispersion fit.

### Morphological and biochemical changes associated with *Gluconacetobacter diazotrophicus* and *Bradyrhizobium japonicum* interaction with rice and soybean

During the period of 120 hai by BRH and GAB, both plant species showed growth significantly higher than that of the control seedling, which corroborated with the earlier observations that these bacteria have Plant Growth Promoting Microbes (PGPM) activity (Chaintreuil et al., 2000; Schneijderberg et al., 2018; Wang et al., 2018; Greetatorn et al., 2019; Bosamia et al., 2020). The growth pattern in rice was much more with GAB than that of BRH, and the reverse was the case in soybean at 120 hai. Soybean and BRH symbiotic association is already known (Couto et al., 2011), but our results revealed that the PGPM activity of this association starts much earlier than the nodule formation and the biological nitrogen fixation. We have studied five important parameters (TRS, MRP, FOLRN, SOLRN, and LRD) of RSA for all of the four (RG, RB, SG, SB) combinations. Almost from the beginning (24 hai) to 120 hai, rice showed a significant increase in all five RSA parameters with GAB inoculation, and soybean showed a similar increase with BRH inoculation. RB and SG also showed an increase in most of the RSA parameters, but significantly less than those in RG and SB. This also establishes that the RG compatibility is better than that of RB and that the SB compatibility is better than that of SG. It can be extended that the bacteria primarily and preferentially promoted root proliferation, especially the crucial lateral roots, which secure the acquisition and transport of plant nutrients from the media (Lynch, 1995).

The root colonization using SEM showed that RG and SB had better root colonization than RB and SG, respectively. It clearly indicated that rice prefers endophytic GAB over symbiotic BRH, whereas soybean, as a legume, is adapted to the formation of root nodule symbiosis and prefers BRH over endophytic GAB. Better root colonization is essential and mostly linked with compatibility and shows plant growth-promoting activity. In our study, the SEM results in combination with other morphological and growth parameters are in confirmation with the four different bacteria–plant combinations, i.e., RG is more compatible than RB and SB is more compatible than SG (Benizri et al., 2001; Ku et al., 2018).

POD is the biochemical marker and indicator of the host–plant interaction. POD is generally linked with the early response of interaction and is also involved in hypersensitive response. So, we thought to include biochemical parameters and support the experiment. The ability to degrade plant cell walls and scavenge reactive oxygen species (ROS) appears to be an essential characteristic for bacterial establishment and successful endophytic colonization (Liu et al., 2017). Hence, the POD activity observed in this study indicated the stress level of seedling and their ROS-scavenging capacity (Singh et al., 2020). We observed that GAB and BRH behave differently with rice and soybean due to possibly different levels of compatibility. However, the activity showed a gradual decrease in RB after 24 hai, and it became constant in soybean from 48 hai onward. At 120 hai, both plants had significantly low POD activity than the control plants. During any plant–microbe interaction, whether it is beneficial, pathogenic, or parasitic, the plant gets alerted and perceives a certain level of stress. However, since soybean is adapted to the symbiotic microbial association in their rhizosphere, its stress level came down to normal very soon, which was gradual in the case of rice. In the end, the seedling perceived that both organisms were beneficial, which was observed by their POD activity below the uninoculated seedling.

### Rice and soybean distinguish symbiont endophyte through varying molecular mechanisms

The DEGs in the case of RB and RG showed a significant number of genes that were related to defense, which was because the rice perceived the interaction of both diazotrophs as infection during the initial stages. When we compared the DEGs between RB and RG, a greater number of defense-related DEGs (PR protein, disease resistance, secondary metabolites, signaling) were upregulated in RB. However, several DEGs related to transporters such as acyl career protein, lipid transfer protein, sugar transporter, and water transfer protein were upregulated in RG (and downregulated in RB), which were related to plant growth promotion. Overall, the expression of these genes in rice was correlated with higher compatibility with GAB than that with BRH. We also found DEGs for the genes that are required for general growth and development of the plants including secondary metabolites, transcription factors, hormones, signaling, and transporters in both diazotrophic interactions but specific to RB and RG, which supports the PGPB activity of both organisms (de Souza et al., 2016).

Nitrate transporters, mainly high-affinity nitrate transporters, are involved in nitrate transport from media to the seedling when nitrate concentration is low (Hsu and Tsay, 2013; Kumar et al., 2022). Interestingly, we found that many of these nitrogen metabolism-related genes were upregulated more in RG than RB in the present study, which also indicated that RG has a better chance to associate with rice if given the required opportunity.

In our study, only two specific transcripts *Os04G0581100* and *Os02G0702300* were differentially expressed in both conditions, i.e., RG/RC and RB/RC. Os04GO581100 (only common DEGs) encodes a defense-related protein, a member of the 2OG-Fe (II) oxygenase superfamily (Van Damme et al., 2008). Plant enzymes with the Fe (II) 2OG dioxygenase domain catalyze the formation of plant hormones such as ethylene, gibberellins, and anthocyanidins, as well as pigments such as flavones (Wei et al., 2021). Os02G0702300 encodes a conserved hypothetical protein, with an unknown function, and was upregulated in RB and downregulated in RG, and this gene can be a candidate for functional validation in the future course of the investigation.

When soybean was inoculated with diazotrophs, the transcriptomes showed several DEGs. Genes related to defense, disease resistance, and stress were upregulated or downregulated; in fact, more genes were downregulated than upregulated. Genes involved in auxin signal transduction or cellulase biosynthesis were also downregulated. This could be interpreted to mean that, initially, both microbes triggered innate immunity, leading to the induction of defense-related genes (Boller and He, 2009; Muthamilarasan and Prasad, 2013). However, very soon (48 hai), the immune response was suppressed and transcription of defense-related genes was reduced to background levels. Interestingly, BRH is in symbiotic association with soybean, but no major nodulin genes were differentially expressed within the period of 5 days (120 hai) after inoculation except for one transcript, *GLYMA01G03470*, which was downregulated in both SG and SB.

POD could be a biochemical marker and indicator of the plant–microbe interaction, although this enzyme is also involved in other processes such as lignin biosynthesis in plant. POD is generally linked with the early response of plant–microbe interaction and is also involved during stresses. Hence, to know whether the specific plant–microbe interactions in our study lead to any stress during early inoculation, we measured the POD activity. Such interactions were not reported earlier, and hence, POD activity was measured as a parameter to assess the course of interaction.

We also selected a few DEGs from RG and RB combinations (**Supplementary Material S11**) and searched them in the RiceXPro V3.0 database (https://ricexpro.dna.affrc.go.jp/) for gene expression profiles in the whole root inoculated with *Magnaporthe oryzae* for 5 days, and we found several genes that are responsive to pathogenic inoculation. Similarly, a few DEGs found in our study in SB combination (**Supplementary Material S12**) were searched in the SoyBase database (https://soybase.org/), and several of them were found responsive to early inoculation with BRH.

### Differential regulation of metabolic pathways in rice and soybean during diazotrophic interaction

Pathway analysis results showed that the major pathways involved within 5 days of inoculation were related to the biosynthesis of secondary metabolites, phenylpropanoids and flavonoids (Thomas et al., 2019; Wiggins et al., 2022). Bag et al. (2022) suggested that secondary metabolites are crucial for plants’ adaptation to new changing environments and biotic and abiotic stress management. Two pathways, *viz*., “Plant pathogen interaction” and “MAPK signaling,” were specific to RG, whereas the pathways related to nitrogen metabolism and plant hormone signaling were specific to RB. These results suggest that in rice, GAB initially induces an immune response, while this is not the case for BRH. Hence, the rice seedling gets the signal during the early interaction with both BRH and GAB. Regarding nitrogen metabolism in the case of RB, it is known that *Bradyrhizobium* is endophytic in the case of all rice genotypes and significantly contributes to the process of nitrogen fixation (Biswas et al., 2000; Piromyou et al., 2015; Piromyou et al., 2017; Okamoto et al., 2021). The pathways related to nitrogen metabolism must have been activated prominently within 5 days of inoculation, which was not observed in RG.

Induction of genes involved in metabolic pathways related to nitrogen metabolism in GAB and BRH interaction with soybean was more in number than in the case of rice. However, the pathways involved in both bacterial inoculations were mostly what we have mentioned in the case of rice except for glutathione metabolism (antioxidant and ROS/stress reliever), amino acid metabolism and protein processing, phagosome-related activities, ATP-binding cassette transporters, phosphatidylinositol signaling system, peroxisome, and nitrogen metabolism. These pathways are mostly legume–diazotroph interaction-specific and needed for the legume seedlings to get activated during the initiation of the symbiotic association. Glutathione metabolism-related pathway is related to ROS-scavenging during diazotrophic interaction and nodulation (Mandon et al., 2021); amino acid metabolism (biosynthesis, transport, and/or degradation) and are often crucial for the establishment and maintenance of an effective nitrogen-fixing symbiosis process, which is intimately interconnected with the metabolism of the plant (Dunn, 2014); nitrogen signaling and metabolism (Carvalho et al., 2014); and symbiosome-related pathways (phagosome). It is reported that the root cells accommodate the rhizobia by the suppression and defunctionalization of their vacuole and also by retargeting some tonoplast proteins to symbiosome (Gavrin et al., 2014). However, there is a report that suggested that the phagosome and symbiosome membranes may form in a similar way (De La Peña et al., 2018). Genes encoding members of a phosphatidylinositol signaling pathway were found to be differentially regulated in our study; phosphatidylinositol signaling also plays a role in legume–rhizobium interactions (Huang et al., 2016).

### Upregulation of defense-related transcripts governs the interaction

The DEGs were analyzed at varying time points after inoculation with both diazotrophs through qRT-PCR. The major DEGs upregulated in rice plants after inoculation with GAB and BRH were low molecular weight PR proteins.

The induction of PR proteins can be suggested as a part of innate immunity. Innate immunity constitutes the first layer of plant immunity. The MAPK signaling was also observed downstream of this induction that shows the activation of innate immunity response. As an initial response, rice and soybean must have induced the expression of PR proteins. Nevertheless, rice and soybean should have recognized the diazotrophs as beneficial through their effector sequence recognition. In symbiotic interactions as well, it is reported that the generation of ROS leads to the expression of symbiotic POD gene (Peleg-Grossman et al., 2009). Similar roles of PR protein-chitinase have been mentioned earlier in soybean inoculated with BRH (Brechenmacher et al., 2008). The expression of thaumatin-like proteins (TLPs) in our study was the highest at 48 hai in RG and at 24 hai in RB and thereafter decreased gradually until 120 hai, indicating the successful early colonization of rice.

The Myb-related protein *OsMYBAS1* (*Os11G0700500*) is one of the largest transcription factor (TF) families involved in many developmental processes, regulation of defense responses to various stresses, hormone signaling, and many metabolic processes in seedlings (Li et al., 2006). Its overall expression in RG was downregulated, and expression analysis of *OsMYBAS1* revealed that it was gradually decreased in RB; however, no trend was observed in RG and the highest expression was recorded at 48 hai. It may be inferred that GAB and BRH would have communication with rice regulated by *OsMYBAS1*, which may have led to the downregulation of this defense protein. We have studied another class of defense protein, thionin. Thionins are small antimicrobial peptides that are involved in plant defense and are also known as toxins for animals, and the seedlings produce them for defense (Asano et al., 2013; Ji et al., 2015).

In both RB and RG, this gene was initially upregulated slightly, and expression reduced gradually with time, indicating the relieving of stress in rice.

From our differential expression results on high-affinity nitrate transport and its partner protein, this can be deciphered that *OsNRT2.2* plays an important role in root growth and development for efficient nitrate uptake and signaling (Xu et al., 2020), and this transporter along with OsNAR2.1 plays a key role in nitrate absorption and translocation, and their overexpression could greatly improve nitrogen uptake (Feng et al., 2011; Liu et al., 2014). The role of rice *OsNRT2.3a* in the transport of ammonium nitrate (NO)_3_ from root to shoot has been described by Tang et al. (2012). *OsNAR2.1: Os02G0595900* shows that both interactions are different with regard to affecting plant partitioning of nitrogenous metabolites. An *NRT2* (*GLYMA13G39850)* gene was differentially expressed and was annotated as NRT2 (Ranamalie Amarasinghe et al., 1998). Furthermore, SoyBase database indicates that it is similar to *AtNRT2.4*, which plays a role in both root and shoot under nitrogen stress (Kiba et al., 2012). So far, we have noticed that GAB and BRH were more compatible with rice and soybean, respectively, and further molecular studies need to be conducted for assessing the complete progression of interaction.

### Pathways responsible for the difference between the two diazotrophic interactions

A large number of ortholog DEGs were found between rice and soybean when they were grown normally without any interaction with diazotrophs (control). The majority of these DEGs were common when inoculated with either of the diazotrophs (83.1% of the DEGs in BRH and 83.5% of the DEGs in GAB inoculation). This is mainly because the gene expression patterns in rice and soybean, which are two completely different plant types, are expected to be different.

However, we observed that DEGs specific to each diazotrophic inoculation showed the difference between the two diazotrophic interactions regardless of plant type. From these orthologous DEGs, we have found the enriched GO terms and then KEGG pathways, which expectedly showed a similar trend recorded in earlier studies (Nuruzzaman et al., 2013). The orthologous KEGG pathways were mostly related to metabolic pathways for general growth and development including carbohydrate, amino acid, fatty acid, protein, nucleotide, vitamin (vitamin-B family) metabolism, synthesis of structural component (glycerophospholipid) signaling, stress management including glutathione metabolism, biosynthesis of secondary metabolites, flavonoid biosynthesis, defense toward microbial inoculation (terpenoid biosynthesis), plant–pathogen interaction, circadian rhythm, regulation of autophagy, phagosome, and Soluble N-ethylmaleimide-sensitive factor activating protein recepto (SNARE) interactions in vesicular transport.

In addition, many of the orthologous KEGG pathways were enriched in rice that we observed in legumes during symbiotic or endophytic interaction with nitrogen-fixing bacteria (phosphatidylinositol signaling, amino acid metabolism and protein processing, phagosome-related, ATP-binding cassette transporters, nitrogen metabolism, etc.). It suggests that rice has certain orthologous genes of legumes necessary for diazotrophic interaction. In rice, we observed that the genes expressed were defense-related. A similar trend of gene expression and pathways was observed in soybean along with additional pathways that were mainly diazotroph–legume-specific.

## Conclusion

Rice and soybean roots inoculated with GAB and BRH displayed significantly higher colonization in RG and SB after 120 hai, ratifying that rice is more compatible with GAB and soybean is more compatible with BRH. Diazotrophs are perceived as foreign organisms, and an innate immune response is initiated in soybean and in rice, but since soybean is a legume, it has the mechanism to form nodules to host specific diazotrophic microbes; a normal state within 48 hai is achieved. However, in the case of rice, this response returns to a normal level gradually at 72 hai. This suggests that the diazotrophs could have suppressed the plant immune response, maybe *via* the effectors. Further experiments are required for studying the whole process. All morphological parameters improved in rice and soybean, confirming the PGP activity of GAB and BRH. The root growth parameters were significantly enhanced in the case of RG and SB combinations, once again reconfirming the legume and cereal diazotrophic compatibility.

The transcriptome study revealed many host–diazotroph-specific differential gene expressions and metabolic pathways. The DEGs functionally relevant to host and diazotroph interaction have been identified, and six DEGs, *viz*., chitinase, brassinosteroid, auxin, MYB, nodulin, and NRT, were common in rice and soybean in both diazotrophic inoculations. Three DEGs, *viz*., nitrate transport accessory protein (NAR), thaumatin, and thionin, were exclusively present in rice. Three DEGs, *viz*., NAC, ABA, and ammonium transporter, were exclusively present in soybean.

The study was carried out to capture the unique events happening in the early stage of inoculation by two diazotrophs. Further analysis of such interactions in later stages of inoculation can provide useful insights on the missing links involved in nitrogen-fixing bacterial sensing and signaling pathway and genes associated with compatibility and colonization of non-leguminous plants. In addition, the interacting transcripts in GAB and BRH while colonizing the host plants, *viz*., rice and soybean, will be worth studying. Dual transcriptomic analysis has the potential to decipher such interacting transcripts of host–microbe association.

## Data availability statement

The datasets presented in this study can be found in online repositories. The names of the repository/repositories and accession number(s) can be found in the article/**Supplementary Material**.

## Author contributions

Conceptualization, PM, RS and LC; Data curation, PM, and RS; Formal analysis, PM, MS, KB, MK, EM, AP, RG and BS; Funding acquisition, PM; Investigation, PM, KB, and GC; Methodology, MS, PM; Project administration, PM, and RS; Resources, PM, RS, and GC; Software, PM, MS, MK and EM; Supervision, PM, RS and LC; Validation, PM, MS, GC, MK, EM and BS; Visualization, PM, AV and SS; Writing—original draft, MS; Writing—review and editing PM, MS, LC, KB, AV, GC, MK, EM, AP, RG, BS, SS, RS. All authors contributed to the article and approved the submitted version.

## Funding

Research work for this study was funded by the Indian Council of Agricultural Research under the project “Biological Nitrogen Fixation in Rice” which is a part of “Incentivizing Research in Agriculture” and also by ICAR-National Institute for Plant Biotechnology'.

## Acknowledgments

The authors would like to acknowledge the support and guidance provided by the Director, ICAR-National Institute for Plant Biotechnology, New Delhi. M.R.S. also thanks the Director of KIIT School of Biotechnology and KIIT University, Bhubaneswar for providing facilities to pursue Ph.D.

## Conflict of interest

The authors declare that the research was conducted in the absence of any commercial or financial relationships that could be construed as a potential conflict of interest.

## Publisher’s note

All claims expressed in this article are solely those of the authors and do not necessarily represent those of their affiliated organizations, or those of the publisher, the editors and the reviewers. Any product that may be evaluated in this article, or claim that may be made by its manufacturer, is not guaranteed or endorsed by the publisher.
