# Supplementary figures and images for: Understanding plant–microbe interaction of rice and soybean with two contrasting diazotrophic bacteria through comparative transcriptome analysis
Source: Front Plant Sci. 2022 Nov 18;13:939395. doi: 10.3389/fpls.2022.939395 (PMC9724235; doi:10.3389/fpls.2022.939395)

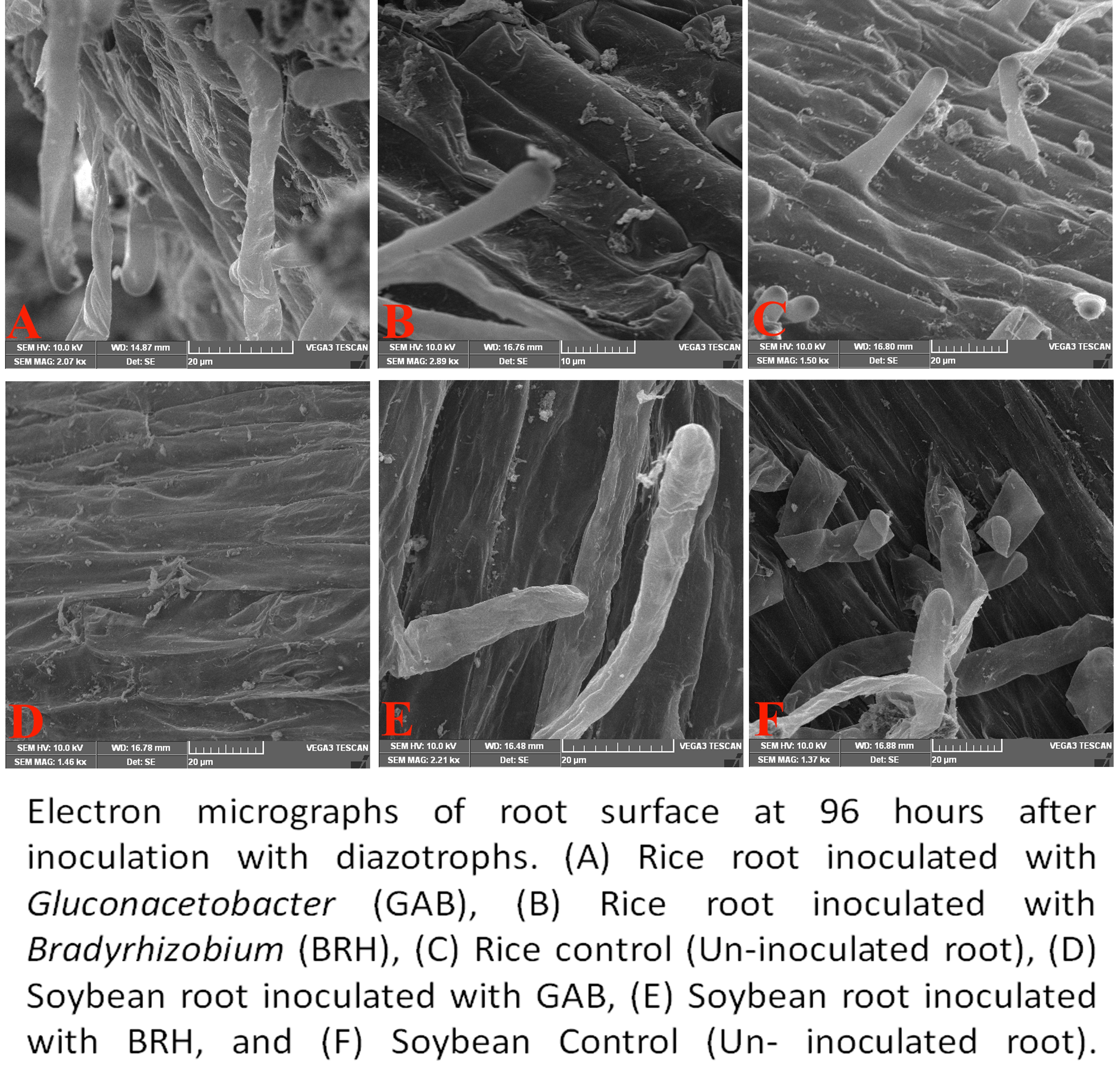

Supplement: Supplementary file 1 [file Image_1.jpeg]

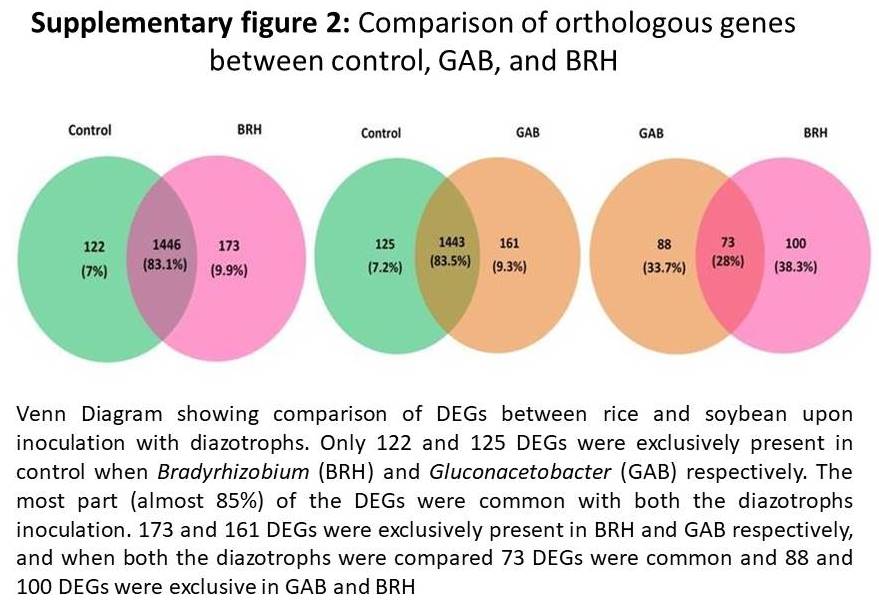

Supplement: Supplementary file 2 [file Image_2.jpeg]
