# Supplementary material for: Understanding plant–microbe interaction of rice and soybean with two contrasting diazotrophic bacteria through comparative transcriptome analysis
Source: Front Plant Sci. 2022 Nov 18;13:939395. doi: 10.3389/fpls.2022.939395 (PMC9724235; doi:10.3389/fpls.2022.939395)
Supplement: Supplementary file 5 [file Table_3.docx]

|  | **Raw_reads** | | | **Clean_Reads** | | | **Total transcript** |
| --- | --- | --- | --- | --- | --- | --- | --- |
|  | **control** | **Gluconacetobacter (GAB)** | **Bradyrhizobium (BRH)** | **control** | **GAB** | **BRH** |  |
| **Rice** | 133589510 | 144948134 | 137524860 | 118294606 | 128147060 | 121726982 | 66220 |
| **Soybean** | 169026252 | 133925160 | 144559410 | 150859976 | 119735702 | 129343902 | 103715 |

**Data statistics of rice and soybean**
