# Supplementary material for: Understanding plant–microbe interaction of rice and soybean with two contrasting diazotrophic bacteria through comparative transcriptome analysis
Source: Front Plant Sci. 2022 Nov 18;13:939395. doi: 10.3389/fpls.2022.939395 (PMC9724235; doi:10.3389/fpls.2022.939395)
Supplement: Supplementary file 6 [file Table_4.docx]

**Complete number of up and down-regulated DEGs in different combinations**

| **Total Transcripts** | | | | |
| --- | --- | --- | --- | --- |
|  | **Total transcripts** | **Total DifferentiallyExpressed Genes (DEG)** | **up-regulated DEGs** | **down-regulated DEGs** |
| **RG_RC** | 22100 | 63 | 38 | 25 |
| **RB_RC** | 21971 | 113 | 20 | 93 |
| **RB_RG** | 22149 | 779 | 201 | 578 |
| **SG_SC** | 34484 | 2509 | 703 | 1806 |
| **SB_SC** | 34644 | 2158 | 808 | 1350 |
| **SB_SG** | 34587 | 3925 | 2305 | 1620 |
| **SC_RC** | 2237 | 1568 | 635 | 933 |
| **SG_RG** | 2227 | 1604 | 645 | 959 |
| **SB_RB** | 2238 | 1619 | 674 | 945 |

**RG- Rice_Gluconacetobacter**

**RC- Rice_Control**

**RB-Rice_Bradyrhizobium**

**SG-Soybean_Gluconacetobacter**

**SC- Soybean _Control**

**SB- Soybean_Bradyrhizobium**
