# Supplementary material for: Understanding plant–microbe interaction of rice and soybean with two contrasting diazotrophic bacteria through comparative transcriptome analysis
Source: Front Plant Sci. 2022 Nov 18;13:939395. doi: 10.3389/fpls.2022.939395 (PMC9724235; doi:10.3389/fpls.2022.939395)
Supplement: Supplementary file 9 [file Table_7.docx]

**Orthologous genes between rice and soybean**.

| Total number of orthologous genes | Differentially Expressed Genes among Soybean Control_Rice Control | DEGs among Soybean Gluconacetobacter_Rice_G | DEGs among Soybean_Bradyrhizobium_Rice_B |
| --- | --- | --- | --- |
| 2238 | 1568 | 1604 | 1619 |
