# Supplementary material for: Understanding plant–microbe interaction of rice and soybean with two contrasting diazotrophic bacteria through comparative transcriptome analysis
Source: Front Plant Sci. 2022 Nov 18;13:939395. doi: 10.3389/fpls.2022.939395 (PMC9724235; doi:10.3389/fpls.2022.939395)
Supplement: Supplementary file 10 [file Table_8.docx]

**Gene Ontology analysis of different combinations of plant-microbe interaction**

|  | **Rice Gluconacetobacter_Rice Control** | **RiceBradyrhizobium_ Rice Control** | **common** | **Soybean Gluconacetobacter_Soybean Control** | **Soybean Bradyrhizobium_Soybean Control** | **common** |
| --- | --- | --- | --- | --- | --- | --- |
| Total number of DEGs involved | 6550 | 12791 |  | 121221 | 121688 |  |
| Total number of GO terms | 8 | 19 | 4 | 232 | 231 | 106 |
| Biological process | 2 | 2 | 1 | 74 | 81 | 34 |
| Molecular function | 4 | 11 | 1 | 122 | 113 | 52 |
| Cellular component | 2 | 6 | 2 | 36 | 37 | 20 |
